# Prevalence and associated determinants of malaria parasites among Kenyan children

**DOI:** 10.1186/s41182-017-0066-5

**Published:** 2017-10-23

**Authors:** Marufa Sultana, Nurnabi Sheikh, Rashidul Alam Mahumud, Tania Jahir, Ziaul Islam, Abdur Razzaque Sarker

**Affiliations:** 1 Health Economics & Financing Research Group, International Centre for Diarrhoeal Disease Research, Bangladesh (icddr,b), 68 Shahid Tajuddin Ahmed Sharani, Mohakhali, Dhaka, 1212 Bangladesh; 2 University of Strathclyde, Glasgow, UK

**Keywords:** Malaria, Children, Prevalence, Odds ratio, Kenya

## Abstract

**Background:**

Approximately 80% of deaths attributed to malaria worldwide occurred mainly in Africa in 2015. Kenya is one of the major malaria endemic countries, making malaria the leading public health concern in this country. This study intended to document the prevalence of malaria and determine associated factors including socioeconomic status among children aged 6 months to 14 years in Kenya.

**Methods:**

This study analyzed the secondary data extracted from the 2015 Kenya Malaria Indicator Survey (KMIS), a cross-sectional country representative survey. Associations of demographic, socioeconomic, community-based, and behavioral factors with the prevalence of malaria in children were analyzed using multivariable logistic regression analysis.

**Results:**

Data from 7040 children aged 6 months to 14 years were analyzed. The prevalence of malaria showed an upward trend in terms of age, with the highest prevalence among children aged 11–14 years. Prevalence was also higher among rural children (10.16%) compared to urban children (2.93%), as well as poor children (11.05%) compared to rich children (3.23%). The likelihood of having malaria was higher among children aged 10–14 years (AOR = 4.47, 95% CI = 3.33, 6.02; *P <* 0.001) compared with children aged under 5 years. The presence of anemia (AOR = 3.52, 95% CI = 2.78, 4.45; *P* < 0.001), rural residence (AOR = 1.71, 95% CI = 1.31, 2.22; *P <* 0.001), lack of a hanging mosquito net (AOR = 2.38, 95% CI = 1.78, 3.19; *P <* 0.001), primary education level of the household head (AOR = 1.15, 95% CI = 1.08, 2.25; *P <* 0.05), and other factors, such as the household having electricity and access to media such as television or radio, were also associated with the likelihood of infection.

**Conclusion:**

This study demonstrated the need to focus on awareness programs to prevent malaria and to use existing knowledge in practice to control the malaria burden in Kenya. Furthermore, this study suggests that improving the information available through the mass media and introducing behavior change communication and intervention program specifically for those of poor socioeconomic status will help to reduce malaria cases.

## Background

Malaria is an entrenched global health challenge and is a major public health concern in many countries including Kenya [1]. It is endemic in over 100 countries, and almost half of the worldwide population is at risk of malaria, where approximately one million people die from malaria each year [2]. This life-threatening disease is transmitted in humans from one person to another indirectly via the bite of female mosquitoes of the genus Anopheles, which harbors one of five species of parasites belonging to the genus Plasmodium [2, 3].

In 2015, malaria transmission had been noted in over 95 countries and territories, constituting approximately 214 million cases. However, approximately 80% of all deaths due to malaria were concentrated in just 15 countries, mainly in the African region [2]. Children and pregnant women are most vulnerable to morbidity and mortality associated with malaria. Globally, approximately 306,000 children under the age of 5 died that year due to malaria, and approximately two thirds of these deaths occurred in the African region [4]. According to the World Health Organization (WHO), it is estimated that 9 out of 10 deaths in children were caused by malaria in Africa [2]. Transmission of malaria highly depends on the temperature, humidity, and rainfall [5]. High temperature and heavy rainfall in summer season leads the highest malaria transmission, especially in Africa [6]. Despite those climatic factors, malaria transmission is also determined by the socioeconomic conditions and knowledge of and access to malaria prevention tools as well as the healthcare services [6]. In Africa, malaria transformation is comparatively higher among the rural setting than urban areas which may be because of the higher vector density, lower housing quality, and the poor drainage systems in rural settings [7]. Malaria is a major threat to public health and is the leading cause of morbidity and mortality in Kenya [8]. Out of 34 million Kenyans, approximately 25 million are estimated to be at risk of malaria, which is more than 70% of the population at risk [6]. An estimated 6.7 million new clinical cases each year, with 4000 deaths occurring particularly among children, make malaria a major health burden for Kenya [9].

Pregnant women and children are high-risk groups for malaria as they are typically affected most severely by this disease, and prevention efforts typically target these vulnerable groups in Kenya [2]. School-aged children (age 5–15) bear the most significant burden of malaria in terms of having the highest prevalence rate [10]. Although a number of studies have been conducted on malaria among children and young adults, most of them are clinical-, treatment-, and prevention-based studies [1, 9, 11].

It is evident that sociocultural context and community attitudes and perceptions have a significant role in prevention and control of malaria cases. However, such studies are rarely examined in the context of Kenya specifically [12, 13]. Furthermore, in the African region, it was demonstrated that community awareness is generally very poor at preventing malaria cases, although it is the cardinal tool currently used for malaria prevention strategies [14]. Greater knowledge, attitudes, and active practices regarding malaria disease are critical in establishing effective control measures. The introductions of mass media and behavior change communication (BCC) to malaria control are well documented and proven interventions which increases the possibility of a better return on malaria programmes [15]. BCC campaigns can create demand among the families to use and hang their nets regularly and can improve malaria prevention as well as treatment behaviors, especially among the vulnerable groups [16]. A study in Kenya regarding the practice of malaria control in a specific division showed above average practices, but approximately 30% of respondents had household members who failed to use the control method properly. That study was based in a specific area and recommended further studies on health care promotion, intervention, and better communication regarding sustainable behavior changes [17]. Another study conducted in Kenya observing the efficacy of text-message reminders found that text-message reminders can increase a child’s compliance with respect to follow-up after anti-malarial treatment [18]. However, the effects of existing knowledge, attitude, and practices (KAP) of Kenyan populations with regard to malaria prevention strategies are rarely examined. Identifying key risk factors by socioeconomic context and incorporating the effect of existing knowledge and practice of malaria prevention are crucial for the effective implementation of prevention and health intervention programs. It is also essential for policy formulation and for the assessment of resource requirements, specifically for low-resource settings, and for intervention prioritization by regions. Furthermore, a better understanding of the association between malaria and sociodemographic factors related to poverty is needed because the financial protection necessary to take remedial measures is a major challenge for some households [9, 11, 12]. Therefore, the intention of this study was to determine the prevalence of malaria among children under 15 years of age, to examine associated determinants considering socioeconomic status, and to examine the effect of knowledge and attitudes concerning malarial disease and its prevention strategies among Kenyan households. The knowledge generated by this study can contribute to the formulation of malaria control programme among the young children of Kenya. Understanding causal association of malaria in sociodemographic context along with the knowledge, attitudes are vital issues which can provide essential insight into malaria burden and helps policy level decision on malaria control strategies.

## Methods

### Data

This study used cross-sectional survey data from a secondary source extracted from the Kenya Malaria Indicator Survey (KMIS), 2015. The survey was based on a nationally representative sample drawn from the four epidemiological zones in Kenya (highland epidemic-prone areas, endemic areas (lake and coast), semi-arid seasonal malaria transmission areas, and low-risk malaria areas) [8]. Data were collected using a two-stage cluster sampling design, based on the sampling frame of the Fifth National Sample Survey and Evaluation Program (NASSEP V), which itself is based on the 2009 Population and Housing Census (PHC) Enumeration Areas (EAs) created by the Kenya National Bureau of Statistics (KNBS) [8]. The sampling frame was divided into four equal sub-samples, from one of which the 2015 KMIS sample data were drawn. In the first stage, a total of 246 clusters (EAs were selected as sample clusters numbering 131 and 115 for rural and urban, respectively) with equal probability of selection were chosen from the NASSEP V master sample. In the second stage, using a systematic sampling technique, a uniform sample of 30 households from each of the selected clusters were selected for the study. The data were collected from 6 July to 15 August 2015, using three types of questionnaires (a Household Questionnaire, a Woman’s Questionnaire, and a Biomarker Questionnaire) that covered a sample of 7313 households based on household surveys executed by the National Malaria Control Programme (NMCP) of the Ministry of Health (MOH) and the Kenya National Bureau of Statistics [8]. From the children aged 6 months to 14 years in the selected households, blood samples were collected for testing anemia and malaria. Hemoglobin analysis was carried out to detect the presence of anemia in the children. Severe anemia was considered to be a hemoglobin level < 8.0 g/dl, and moderate anemia was between 8.0 and 9.9 g/dl. Other anemia classifications varied by age group as follows: children 6–59 months: mild anemia 10.0–10.9 g/dl, no anemia > 11.0 g/dl; children 5–11 years: mild anemia 10.0–11.4 g/dl, no anemia > 11.5 g/dl; children 12–14 years: mild anemia 10.0–11.9 g/dl, no anemia > 12.0 g/dl [8]. Since microscopic examination is the gold standard for the diagnosis of malaria, for this study children were considered as malaria positive or negative based on the result of this test only [19–21]. Approval was obtained from the Demographic Health Survey (DHS) website to use the 2015 KMIS data. A total of 7040 children aged 6 months to 14 years were analyzed for the current study.

### Statistical analysis

All statistical analyses were performed using the statistical package Stata/SE 13.0 and significant associations have been measured at 5% alpha level (*p* < 0.05). Based on the MIS instruction, sampling weight was used for cluster adjustment. Both bivariate and multivariable statistical analyses were conducted during data analysis. Bivariate analysis was carried out to explore the prevalence of malaria compared to different selected variables and to the knowledge and the attitude of the respondent. The chi-squared test of independence was used to determine any significant associations between positive blood smear test results and attitudes, knowledge, and measures in terms of the *P* value. Based on significant associations with the results, variables were chosen for the multivariate analysis [6]. Binary logistic regression model was used to trace the significant determinants for malaria, and the results were presented in terms of odds ratio (OR) that controlled for multiple confounders (with 95% confidence interval). A binary logistic regression model was used in this analysis because the outcome variable has a binary response of malaria positive or negative. This outcome variable was re-coded as “0” for children who did not have malaria and “1” for those who had malaria. Both adjusted and unadjusted ORs were considered for finding the single and multi-factorial (covariates) effects in the model [22]. Age, sex, and anemia levels of children, sex, and education of household head, household electricity status, media exposure, number of living room, net hanging status for sleeping, residence, and wealth index were used as independent variables in multivariate regression model. Some independent variables were used as per the original dataset and some were re-coded depending on research interests. Socioeconomic status was measured by a wealth index, which is a composite measure of a household’s cumulative living standard calculated using data on the household’s selected assets by generating a weight or factor score through principal components analysis [8].

## Results

### Prevalence of malaria

Malaria prevalence increases with increasing age of the children in this population. Comparatively aged children (10–14 years) suffer more due to malaria in terms of the prevalence (10.22%), whereas malaria prevalence was 4.83% among children under the age of 5 (Table 1). Malaria prevalence was considerably higher among male children (8.23%) than female children (8.04%).

**Table 1:** Distribution of the prevalence malaria among 6-month to 14-year children on sociodemographic characteristics (*N* = 9040)

Variable	*N* (%)	Prevalence of malaria *n* (%)	95% CI for prevalence of malaria
Age of children			
0.5–4.9 years	2971 (32.87)	144 (4.83)	(4.11, 5.66)
5–9 years	3334 (36.88)	313 (9.38)	(8.44, 10.42)
10–14 years	2735 (30.25)	279 (10.22)	(9.14, 11.41)
Sex of children			
Male	4590 (50.78)	378 (8.23)	(7.47, 9.06)
Female	4450 (49.22)	358 (8.04)	(7.28, 8.88)
Anemia levels of children			
Anemic	1727 (19.11)	292 (16.92)	(15.23, 18.77)
Not anemic	7313 (80.89)	443 (6.06)	(5.54, 6.63)
Sex of household head			
Male	5942 (65.73)	537 (9.04)	(8.34, 9.79)
Female	3098 (34.27)	199 (6.41)	(5.60, 7.33)
Education of household head			
No education	1493 (16.52)	117 (7.82)	(6.56, 9.30)
Primary	4549 (50.33)	500 (10.98)	(10.10, 11.92)
Secondary and higher	2998 (33.16)	119 (3.98)	(3.34, 4.75)
Household has electricity			
No	6787 (75.08)	695 (10.24)	(9.54, 10.99)
Yes	2253 (24.92)	41 (1.80)	(1.33, 2.44)
Media exposure			
Household has television			
No	6494 (71.84)	676 (10.40)	(9.68, 11.17)
Yes	2546 (28.16)	60 (2.36)	(1.84, 3.03)
Household has radio			
No	2993 (33.11)	299 (9.99)	(8.97, 11.12)
Yes	6047 (66.89)	437 (7.22)	(6.60, 7.90)
No. of living room			
One	3456 (38.23)	257 (7.43)	(6.60, 8.35)
Two	3559 (39.37)	356 (10.00)	(9.05, 11.03)
Three and more	2024 (22.41)	123 (6.08)	(5.12, 7.21)
Net is hanging for sleeping (*N* = 4458)			
Not hanging	352 (7.89)	81 (23.11)	(18.99, 27.81)
Hanging	4106 (92.11)	330 (8.05)	(7.25, 8.92)
Residence			
Urban	2527 (27.96)	74 (2.93)	(2.34, 3.67)
Rural	6513 (72.04)	662 (10.16)	(9.45, 10.92)
Wealth index			
Poor	4054 (44.85)	448 (11.05)	(10.12, 12.05)
Middle	1903 (21.05)	188 (9.90)	(8.64, 11.33)
Rich	3083 (34.11)	99 (3.23)	(2.66, 3.91)

**Fig. 1:**
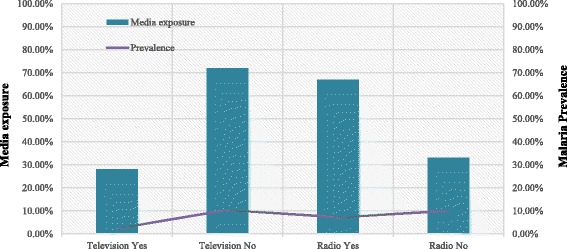
Prevalence of malaria among media exposure and non-exposure households

Malaria prevalence varies between urban and rural areas, with children from rural areas having a higher prevalence (10.16%) compared to children from urban areas (2.93%). Among all children, approximately 19% were found to be anemic, and malaria prevalence was more than two times higher among these children (16.92%, 95% CI = 15.23, 18.77) compared to non-anemic children (6.06%, CI = 5.54, 6.63). Education of the household head appeared to be an important factor to control malaria among children since malaria cases were higher among children with less-educated (10.98%) and illiterate (7.82%) household heads. In this analysis, approximately 92.11% of the respondents reported using a mosquito net for sleeping, and malaria prevalence was observed to be more than two times higher among households that did not use mosquito nets (23.11%) compared to net user households (8.05%). Our study reveals a higher malaria prevalence (11.05%) among children from poor communities, followed by children from middle class (9.09%) and rich (3.23%) communities. Furthermore, malaria prevalence was higher in children whose household did not have access to media, such as radio and television, with prevalence rates of 9.99 and 10.40% for not having radio or television compared to 7.22 and 2.36% in those having access to radio or television, respectively (Fig. 1).

### Knowledge and attitude towards malaria

The knowledge and attitudes of the study respondents regarding malaria are presented in Table 2. According to this, approximately 69.76% of the study respondents remarked that hanging a mosquito net is extremely important for preventing malaria while only 1.24% reported that it is not important at all. However, the malaria prevalence was higher for children in those households (9.70%) who mention that the hanging net is extremely important, showing variation between knowledge and practice. In terms of the ‘importance of having children sleep under a treated net’, 75.02% of the respondents regarded it as extremely important, where 24.86% regarded it as just important. However, the prevalence was higher among the households’ children whose respondents answered that a treated net was extremely important (9.61%) compared to those who answered just important (4.74%). Approximately 67% of the respondents agreed that people in the community should sleep under an insecticide-treated net (ITN) all the time while 32% disagreed with this statement. The children of respondents that disagreed had the lowest malaria prevalence (4.10%), with a prevalence of 10.24% for those who agreed. Higher malaria prevalence was found (8.84%) among the children whose household respondents disagreed with the risk of malaria in the rainy season than among those who agreed (7.19%).

**Table 2:** Chi-square test of association on respondent’s knowledge, attitude on malaria and mosquito net (*N* = 9040)

Variable	*N* (%)	Prevalence of malaria *n* (%)	*P* value
Importance of hanging a mosquito net to reduce malaria			
Extremely important	6307 (69.76)	652 (9.7)	< 0.01
Important	2622 (29.00)	169 (5.36)	
Not at all	112 (1.24)	9 (6.77)	
Importance of having children sleep under a treated net			
Extremely important	6782 (75.02)	703 (9.61)	< 0.01
Important	2247 (24.86)	127 (4.74)	
Not at all	11 (0.12)	–	
Frequency of using net other than sleeping			
All the time	202 (2.24)	14 (6.93)	< 0.01
Sometimes	1697 (18.77)	219 (9.87)	
Never	7141 (78.99)	597 (7.87)	
Treated nets are safe for sleeping			
Agree	8725 (96.51)	809 (8.35)	0.31
Disagree	315 (3.49)	21 (6.73)	
Most people in the community sleep under a ITN all the time			
Agree	6080 (67.25)	700 (10.24)	< 0.01
Disagree	2960 (32.75)	130 (4.10)	
A net can be hanged everywhere in the house			
Agree	8003 (88.53)	777 (8.75)	< 0.01
Disagree	1037 (11.47)	53 (4.71)	
Risk of malaria is higher in rainy season			
Agree	2577 (28.51)	236 (7.19)	< 0.01
Disagree	6463 (71.49)	594 (8.84)	

### Determinants of malaria

Logistic regression models were constructed to examine sociodemographic determinants of malaria prevalence and are reported in Table 3. The age of the children, presence of anemia, education of the household head, household having electricity, access to television, residence type (rural or urban), and mosquito net use behavior for sleeping were found as significant determinants for malaria in children by both models, and the effects of these variables were directly predicted using the odds ratio [23]. From model II, the child’s age was one of the most significant factors for malaria among children aged 6 months to 14 years. The odds ratio of children aged 5–9 years (OR = 2.29, 95% CI = 2.23, 3.82) and 10–14 years (OR = 4.47, CI = 3.33, 6.02) demonstrated that these two age groups were more vulnerable to malaria than children under 5 years (reference group). Anemic children were 3.52 times more likely to have malaria compared to their non-anemic counterparts (OR = 3.52, 95% CI = 2.78, 4.45). The analysis of odds ratios also verified that children from households with uneducated heads (OR = 1.15, CI = 1.08, 2.25) and primary educated heads (OR = 1.82, CI = 1.35, 2.44) were more prone to have malaria than children of secondary and higher educated household heads. The children of households having no electricity were also more likely to have malaria (OR = 3.08, CI = 1.77, 5.34) compared to those in households with access to electricity. It was also found that the probability of having malaria was more likely among rural children than urban children (OR = 1.17, CI = 1.31, 2.22) and for the children of households with no access to television (OR = 1.63, CI = 1.01, 2.63) than their counterparts. Children of the households not using a bed net for sleeping were 2.38 times more susceptible to malaria (OR = 2.38, CI = 1.78, 3.19) compared to net users.

**Table 3:** Multivariable logistic regression model on sociodemographic determinants of malaria

Variable	Model I	Model II
	Unadjusted OR (95%CI)	Adjusted OR (95%CI)
Age of children		
Under 5 years (ref)	1.00	1.00
5–9 years	1.89*** (1.56, 2.29)	2.92*** (2.23, 3.82)
10–14 years	2.23*** (1.83, 2.70)	4.47*** (3.33, 6.02)
Sex of children		
Male	1.05 (0.91, 1.21)	1.09 (0.89, 1.33)
Female (ref)	1.00	1.00
Anemia levels of children		
Anemic	2.86*** (2.46, 3.32)	3.52*** (2.78, 4.45)
Not anemic (ref)	1.00	1.00
Sex of household head		
Male	1.24*** (1.06, 1.45)	1.18 (0.95, 1.47)
Female (ref)	1.00	1.00
Education of household head		
No education	1.94*** (1.53, 2.46)	1.56** (1.08, 2.25)
Primary	2.80*** (2.29, 3.43)	1.82*** (1.35, 2.44)
Secondary and higher (ref)	1.00	1.00
Household has electricity		
No	5.67*** (4.17, 7.71)	3.08*** (1.77, 5.34)
Yes (ref)	1.00	1.00
Media exposure		
Household has television		
No	4.80*** (3.66, 6.31)	1.63** (1.01, 2.63)
Yes (ref)	1.00	1.00
Household has radio		
No	1.46*** (1.27, 1.69)	1.08 (0.86, 1.36)
Yes (ref)	1.00	1.00
No. of living room		
One	1.09 (0.89, 1.33)	1.08 (0.81, 1.45)
Two	1.44** (1.19, 1.75)	1.29** (0.99, 1.69)
Three and more (ref)	1.00	1.00
Net is hanging for sleeping		
Not hanging	3.65*** (2.80, 4.75)	2.38*** (1.78, 3.19)
Hanging (ref)	1.00	1.00
Residence		
Urban (ref)	1.00	1.00
Rural	2.54*** (2.13, 3.03)	1.71*** (1.31, 2.22)
Wealth index		
Poor (lowest 40%)	3.67*** (2.93, 4.59)	1.12 (0.73, 1.72)
Middle (40%)	3.08*** (2.38, 3.98)	1.04 (0.68, 1.58)
Rich (ref)	1.00	1.00

**p < 0.05, ***p < 0.01

Variable included in the multivariate model (model II): malaria test result either test positive (coded 1) or test negative (coded 0) for both model I and model II. Age, sex, and anemia levels of children, sex and education of household head, household electricity status, media exposure, number of living room, net hanging status for sleeping, residence, and wealth index were used as independent variables in multivariate regression model (model II)

## Discussion

This study identified the prevalence and examined sociodemographic and knowledge-based factors that determine the likelihood of malaria infection among children aged 6 months to 14 years in Kenya based on country representative secondary data from the 2015 Kenya Malaria Indicator Survey (KMIS). The government of Kenya tries to ensure improved health service delivery with a high priority for malaria prevention and control [11]. The government has developed several effective strategies for monitoring and evaluating malaria control on a regular basis, mainly focused on the reduction of malaria morbidity and mortality by 2018 [11]. This study enables understanding of the association between malaria and sociodemographic factors, revealing that factors such as age, education, economic status of the household, media access, knowledge, and attitude have potential impacts for affecting the prevalence of malaria among the Kenyan children.

This research exposed that malaria prevalence was lower among children less than 5 years old, and susceptibility tends to increase along with increasing age. This is consistent with the finding of another study in Africa, where malaria prevalence was found to be higher among children aged 5–18 years [24]. Male children were found to have a higher prevalence than female children. Several studies also reported similar findings, which may be due to female children being less biologically vulnerable to infectious diseases than male children [1, 10]. The significantly higher prevalence of malaria among the anemic children was also in line with other studies concerning malaria [20, 25].

Higher prevalence was also found among the children whose household heads were less educated. This might be because higher educated heads of household could take more protective measures to reduce exposure that would prevent malaria infection [1]. Similar to other studies, this study also revealed that rural children experienced more malaria cases than urban children, which might be due to less availability of health care facilities and lack of proper social mobilization concerning malaria prevention [26]. Malaria prevalence was also higher among poor children than their rich counterparts. A previous study shows that people in poor households in Kenya generally sleep on the floor and are more vulnerable to be infected with malaria. Additionally, because of their low economic means, they are not able to bear the expenses associated with taking preventive action against malaria [10]. Malaria is also well acknowledged as a disease of poor communities because of their vulnerability and decreased financial means to buy malaria control tools [12]. Similar to an earlier study, using a net could be an effective preventive measure against malaria among children in Kenya [27]. The interesting findings noted here concerning the knowledge and attitude about malaria prevention among Kenyans show a contradictory relationship between knowledge and prevalence of malaria. There was a higher percentage of malaria occurrences among the members of households with higher knowledge regarding malaria prevention, revealing a gap between knowledge and practices. A lack of practice of both indoor and outdoor vector control measures is strongly related to higher malaria prevalence [13]. Another study revealed that although participants had knowledge about prevention strategies against malaria, it was rarely seen in their practices, which supported the significant differences between knowledge and practices of malaria prevention [28]. One study addressed the reasons behind not using mosquito nets, with discomfort (primarily due to heat) being the most reported reason by participants [27]. The contradictory results which may be because of the weak association of malaria knowledge with the use of bed nets and ITNs [29]. Additionally, other socioeconomic and household factors may be responsible for this contradictory relationship between knowledge and inconsistent behavior which leads higher prevalence of malaria [29, 30].

Preventive education campaigns are recommended focusing on the translation of knowledge into practices [13]. Reinforcement of good protective vector control behavior is needed in these circumstances [2]. This study found that media played an important role in the prevention of malaria as its prevalence is lower among those who have watched television or listened to radio programs addressing malaria intervention programs, which supports the positive findings of the influence of mass media for eliminating malaria in African settings [31].

There are several limitations of the study. The study used data from a secondary source based on a cross-sectional design and thus had limited opportunities to measure any causal association between malaria and other factors. Information collected from respondents was self-reported and might be affected by recall bias when highlighting knowledge, perception, and practices. Microscopy test may affect the prevalence of malaria, especially in endemic populations with the low transmission of infection [20, 21, 32]. Despite these limitations, this study generates distinctive information regarding determinants of malaria from country representative data, which could be helpful for formulating further steps to implement interventions.

## Conclusion

Findings of this study revealed that malaria still remains a public health problem, especially for children under 15 in Kenya. It also demonstrated some significant risk factors with independent effects on the prevalence of malaria among Kenyans. This study also found a gap in translating knowledge into practice to prevent the potential infections. However, improvements in these factors with proper practice of preventive measures might have a positive effect in reducing malarial infection. Based on the findings in the present study, multi-modal programs are needed to control malaria in Kenya. Furthermore, need-based innovative interventions and introducing Behavior Change Communication program (BCC) to prevent and treat malaria are recommended to reduce the health burden caused by malaria. Education and awareness programs are suggested to use existing knowledge in practice to control malaria. Communications should be employed by a combination of radio and television programs, posters at local health facilities or identified public places, the formulation of groups of local stakeholders, and interventions such as the distribution and use of insecticide-treated mosquito nets, especially for households with poor socioeconomic status. These interventions are strongly suggested for prevention of malaria cases in Kenya. This study also suggests taking actions towards income-generating interventions (e.g., poultry raising, farming) among the rural poor community, which can improve their financial means to buy safety measures to control malaria.

## Abbreviations

AOR: Adjusted odds ratio
BCC: Behavior change communication
CI: Confidence interval
DHS: Demographic Health Survey
EAs: Enumeration areas
icddr,b: International Centre for Diarrhoeal Disease Research, Bangladesh
ITN: Insecticide-treated net
KAP: Knowledge, attitude, and practices
KMIS: Kenya Malaria Indicator Survey
KNBS: Kenya National Bureau of Statistics
MOH: Ministry of Health
NASSEP: National Sample Survey and Evaluation Program
NMCP: National Malaria Control Programme
OR: Odds ratio
PHC: Population and Housing Census
RDT: Rapid diagnostic test
SES: Socioeconomic status
WHO: World Health Organization
